# Interactions Between Endosymbionts *Wolbachia* and *Rickettsia* in the Spider Mite *Tetranychus turkestani*: Cooperation or Antagonism?

**DOI:** 10.3390/microorganisms13030642

**Published:** 2025-03-12

**Authors:** Sha Wang, Xinlei Wang, Ali Basit, Qiancheng Wei, Kedi Zhao, Yiying Zhao

**Affiliations:** College of Agriculture, Shihezi University, Shihezi 832003, China; 20222012067@stu.shzu.edu.cn (S.W.); 20222012077@stu.shzu.edu.cn (X.W.); basitali27297@stu.shzu.edu.cn (A.B.); 13002637216@163.com (Q.W.); zhaokedibs@163.com (K.Z.)

**Keywords:** endosymbionts, *Rickettsia*, *Wolbachia*, interactions, *Tetranychus turkestani*

## Abstract

Maternally inherited endosymbionts are widespread in arthropods, with multiple symbionts commonly co-existing within a single host, potentially competing for or sharing limited host resources and space. *Wolbachia* and *Rickettsia*, two maternally-inherited symbionts in arthropods, can co-infect hosts, yet research on their combined impacts on host reproduction and interaction remains scarce. *Tetranychus turkestani* (Acari: Tetranychidae) is an important agricultural pest mite, characterized by rapid reproduction, a short life cycle, and being difficult to control. *Wolbachia* and *Rickettsia* are two major endosymbiotic bacteria present in *T. turkestani*. This study used diverse parthenogenetic backcross and antibiotic screening to explore the reproductive effects of these two symbionts on *T. turkestani*. The results show that single *Rickettsia* infection induced male killing in the amphigenesis of *T. turkestani*, leading to arrhenotokous embryo death and fewer offspring. Single *Wolbachia* infection induced strong cytoplasmic incompatibility (CI). During dual infection, CI intensity decreased because *Rickettsia*’s male-killing effect antagonized the *Wolbachia*-induced CI. Dual-infected mites had increased oviposition, lower mortality, a higher female-to-male ratio, and more offspring, thus enhancing *T. turkestani*’s fitness. These findings will be helpful for understanding the nature of host–endosymbiont interactions and the potential for evolutionary conflicts, offering insights into their co-evolutionary relationship.

## 1. Introduction

Endosymbiotic bacteria are common in arthropods, with over 50% of species infected, primarily via maternal inheritance [1,2]. Endosymbiotic bacteria have co-evolved with their hosts, influencing their nutrition, digestion, resistance, and defense against predators, and thereby playing critical role in host colonization and ecological evolution in specific habitats [3,4]. At present, the most studied secondary endosymbionts, including *Wolbachia*, *Cardinium*, *Rickettsia*, and *Spiroplasma*, are known to manipulate the host reproductive and developmental processes by inducing cytoplasmic incompatibility, male killing, parthenogenesis, heat resistance, and drug resistance in their hosts [5,6,7,8].

*Wolbachia* is a maternally transmitted Gram-negative bacteria found in arthropods. The host range of *Wolbachia* is extremely wide, and approximately 65% of insect species naturally carry this endosymbiont. *Wolbachia* is abundantly present in insect ovaries and testes and is also distributed in non-reproductive tissues, such as the head, muscles, midgut, salivary gland, Malpighian tubules, hemolymph, and fat body of insects [9,10]. The regulatory effects of *Wolbachia* on its host have always been a hot topic in *Wolbachia*-related research. Currently, the documented reproductive regulations of the host by *Wolbachia* include cytoplasmic incompatibility (CI), male killing, feminization, parthenogenesis, etc. CI is the most common reproductive regulation induced by *Wolbachia*, which refers to the phenomenon where mating between a *Wolbachia*-infected male and an uninfected female insect results in either no or few offspring. The parthenogenesis induced by *Wolbachia* in *T. turkestani* is arrhenotokous parthenogenesis; that is, the oocytes of female individuals develop directly into new individuals without the fertilization process, and the haploid eggs develop into male offspring [11]. In addition, some strains of *Wolbachia* can also affect the host’s sense of smell, lifespan, immunity, nutrition, fertility, developmental processes, etc. [9,10,12,13,14,15,16,17].

*Rickettsia* is an intracellular symbiotic bacterium that spreads and causes diseases in humans and animals. It is also a secondary endosymbiont that exists in insects. *Rickettsia* is a Gram-negative bacterium that belongs to the family *Rickettsiaceae* in the α subgroup of *Proteobacteria*. It is widely distributed in nature, and its hosts include vertebrates, arthropods, annelids, amoebas, ciliates, hydrozoans, and plants [7]. Research has found that *Rickettsia* and its host insects share a mutualistic symbiotic relationship and have co-evolved. *Rickettsia* can affect the reproductive behavior of their host by inducing male killing and parthenogenesis, and it also has an impact on the fitness of the host insects [8,16,17,18,19].

In nature, co-infection of arthropod hosts by different symbiotic bacteria is quite common. The impacts of multiple infections on the host may be cumulative [20]. The interactions among co-infecting symbiotic bacteria may lead to reproductive phenotypes that are completely different from those seen in singly infected hosts. If co-infection confers a higher fitness than a single infection, it can be stably maintained within the host population [21]. *Rickettsia* and *Wolbachia* sometimes co-infect arthropods. However, little research has been conducted on the interactions between these two bacteria [2,22,23,24,25], and studies on co-infections have only focused on the expression of cytoplasmic incompatibility, while the impact of co-infection of these two bacteria on host reproduction has not been reported.

**Figure 1:**
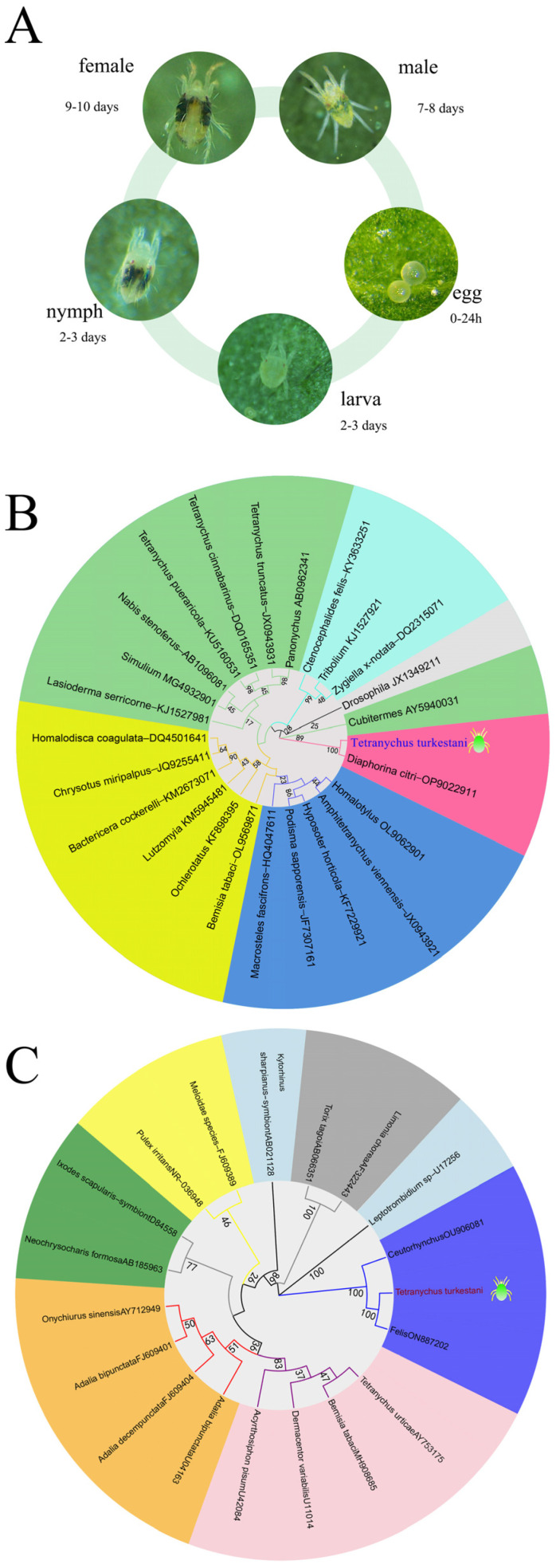
Schematic diagram of the life cycle and phylogenetic trees. (**A**) Schematic diagram of the life cycle of *Tetranychus turkestani*. (**B**) *Wolbachia*. (**C**) *Rickettsia*. The numbers at the nodes are bootstrap values, which were used to evaluate the reliability of the branching structure.

*Tetranychus turkestani* (Ugarov et Nikolski) is an important agricultural pest mite that is distributed throughout Russia, Kazakhstan, the United States, and the Middle East region. Currently, in China, it is only found in Xinjiang [26,27]. Xinjiang is the largest cotton-growing area in the world. *T. turkestani* reproduces rapidly and has a short generation cycle, making it the dominant population of pest mites in cotton fields in Xinjiang. *T*. *turkestani* has five developmental stages, including egg, larva, protonymph, deutonymph, and adult (Figure 1A). The host plants of *T*. *turkestani* include more than 150 species belonging to 25 families. It harms the host plants by sucking their sap. In the early stage of damage, light yellow spots will appear on the front side of the leaves of the damaged plants, and when the damage is severe, purplish red patches will form. In the later stage of damage, the leaves of the plants will wrinkle and deform, finally drying up and falling off [28]. Investigations on cotton fields in different regions of Xinjiang have found that various endosymbiotic bacteria exist in *T. turkestani*, including *Wolbachia*, *Cardinium*, *Rickettsia*, *Spiroplasma*, etc., and the densities of the endosymbiotic bacteria are also different in different geographical regions [29]. In this study, we compare the different hybridization types of four different infected strains of *T. turkestani* (double-infected *Rickettsia* and *Wolbachia* strain I_WR_, single-infected *Rickettsia* strain I_R_, single-infected *Wolbachia* strain I_W_, and double-uninfected strain I_U_), and investigate the effects of *Wolbachia* and *Rickettsia* on the host and their interaction. These results will further enhance our understanding of the reproductive manipulation induced by the co-infection of symbiotic bacteria in arthropods.

## 2. Materials and Methods

### 2.1. Collection and Rearing of Spider Mites

*Tetranychus turkestani* were collected in 2019 from the experimental field of the College of Agriculture, Shihezi University. Then, they were reared in a light incubator at the Insect Physiology Laboratory of the College of Agriculture, Shihezi University, under controlled conditions (25 °C, a photoperiod of 16 h of light and 8 h of darkness, and relative humidity of 60%) [30]. The mites were fed on *Phaseolus vulgaris* L. throughout the rearing process, with no exposure to any pesticides.

### 2.2. Detection of Infections Using Different Symbiotic Bacteria

Extraction of total DNA: First, 25 μL of STE buffer (100 mmol/L NaCl, 10 mmol/L Tris-HCl, 1 mmol/L EDTA, pH = 8.0) was added to a 1.5 mL centrifuge tube. A single spider mite was picked with an insect needle and placed in the tube, and then thoroughly crushed with a plastic pestle. Subsequently, 2 μL of proteinase K (10 mg/mL) was added [30]. The mixture was centrifuged at 3000 r/min for 2 min, incubated at 37 °C for 30 min, heated at 95 °C for 5 min, and centrifuged again at 3000 rpm for 2 min. Finally, 2 μL of the supernatant was used as the template for PCR amplification.

Primers were designed using Beacon Designer 7 software to detect whether *T*. *turkestani* was infected with *Wolbachia* and *Rickettsia* (see Supplementary Materials, Tables S2 and S5).

### 2.3. Establishment of Strains Infected with Different Endosymbiotic Bacteria

Establishment of a strain co-infected with *Wolbachia* and *Rickettsia*: A complete and fresh kidney bean leaf was put in a Petri dish with a sponge (9 cm diameter) and divided into four approximately equal chambers using moistened cotton strips according to the leaf size [30]. Unmated female mites were selected in the static III state from the laboratory strain and were placed individually into each chamber for parthenogenesis. When the offspring developed into adult male mites, the mother was backcrossed with her male offspring. After two days of backcrossing, the mother was transferred to a new chamber for oviposition. After seven days, PCR was performed to detect the mother. These steps were repeated for five generations with the offspring of the female mites with co-infection of *Wolbachia* and *Rickettsia*, and then 30 of them were selected for PCR detection of the infection rates of *Wolbachia* and *Rickettsia*. Once all were infected, a strain co-infected with *Wolbachia* and *Rickettsia* was obtained.

Experimental strains with a single infection of *Rickettsia* and a single infection of *Wolbachia* were obtained using the same method.

Establishment of a completely uninfected strain of *T. turkestani*: A complete and fresh kidney bean leaf was soaked in a 0.2% tetracycline solution for 24 h and then placed into a 9 cm diameter Petri dish with a sponge. Moist cotton strips were placed around the bean leaf to prevent the spider mites from escaping. Newly hatched *T. turkestani* larvae (unfed, nearly white) were selected and placed on the leaf, where they were allowed to grow and reproduce naturally. Distilled water was added daily to the Petri dish to maintain the moisture of the sponge, and the leaf was replaced with a fresh one in a timely manner. Once the larvae matured, about 30 individuals were selected for PCR detection of *Wolbachia* and *Rickettsia* infections. If no infection was detected, the offspring of this strain were continuously cultured to obtain an experimental strain uninfected by *Wolbachia* and *Rickettsia*.

Nomenclature of spider mite strains: I_W_ represents the strain singly infected with *Wolbachia*, I_R_ represents the strain singly infected with *Rickettsia*, I_WR_ represents the strain co-infected with *Wolbachia* and *Rickettsia*, and I_U_ represents the uninfected strain. F stands for female, and M stands for male.

### 2.4. Wolbachia and Rickettsia Phylogenetic Tree Construction

The *wsp* sequence of *Wolbachia* and the *gltA* sequence of *Rickettsia* (see Supplementary Materials, Tables S2–S4) were used in the PCR [31]. PCR amplification products were detected using 1% agarose gel electrophoresis, and positive results were further purified using gel recovery. Then, the purified products were sent to Youkang Biotechnology Co., Ltd. for bidirectional sequencing. Sequences of *Wolbachia wsp* and *Rickettsia gltA* from different species were searched and downloaded from the NCBI database. ClustaIW sequence alignment was performed using MEGA11, and an NJ (neighbor-joining) phylogenetic tree was constructed. Bootstrap analysis with 1000 replicates was conducted.

### 2.5. Detection of the Maternal Inheritance Efficiency of Wolbachia and Rickettsia

The maternal inheritance efficiency of the symbiotic bacteria *Wolbachia* and *Rickettsia* was determined by measuring the infection rates of the two bacteria in the male offspring from the parthenogenesis of single female mites or the female offspring from the sexual reproduction of single pairs of *T. turkestani* [31,32]. The parthenogenetic offspring of I_W_, I_R_, and I_WR_ female mites, the bisexual reproductive offspring of I_W_ female mites and I_U_ male mites, the bisexual reproductive offspring of I_R_ female mites and I_U_ male mites, and the bisexual reproductive offspring of I_WR_ female mites and I_U_ male mites were selected. Using the primers for *Rickettsia gltA* and *Wolbachia wsp*, the infection statuses of *Rickettsia* and *Wolbachia* were detected by PCR. A total of 10/50 female mites were randomly selected (since the number of parthenogenetic offspring of I_R_ female mites was relatively small, 50 I_R_ female mites were selected) to determine whether they underwent arrhenotokous parthenogenesis or bisexual reproduction. Subsequently, 10/2 male or female offspring from each female mite were tested, with a total of 100 offspring individuals in each group. Based on the PCR amplification results, the infection rates of *Wolbachia* and *Rickettsia* were calculated.

### 2.6. Detection of the Titers of Wolbachia and Rickettsia in T. turkestani

Based on the *gltA* gene sequence of *Rickettsia* and the *wsp* gene sequence of *Wolbachia*, specific quantitative primers were designed to detect the titers of *Rickettsia* and *Wolbachia* in *T*. *turkestani*. The *RPSI8* reference gene was selected as an internal control for data standardization and quantification [33] (see Supplementary Materials, Tables S5–S7). Adult male and female mites from different infected strains were quantified, with 200–300 individuals per group constituting one replicate, and the experiment was repeated three times. Quantitative PCR (qPCR) reactions were performed on an ABI Prism 7500 qPCR instrument. The PCR cycling conditions were as follows: 95 °C for 30 s; 95 °C for 5 s; 60 °C for 30 s; 40 cycles. To verify the specificity of the qPCR products, a melting curve (95 °C for 15 s; 60 °C for 1 min; 95 °C for 15 s) was conducted at the end of the reaction. Three technical replicates were performed for each sample. A negative control was set for each reaction. The titer data of *Wolbachia* and *Rickettsia* in *T*. *turkestani* were analyzed using SPSS 26.0, and the expression levels were calculated by the 2^−ΔΔCt^ method. Statistical significance analysis was performed using Student’s *t*-test.

### 2.7. Effects of Different Symbiotic Bacteria Infections on the Fecundity of T. turkestani

Parthenogenesis: Fresh kidney bean leaves were used, and each leaf was divided into four circular sections with an area of approximately 4 cm^2^ each. Single female mites in the static III stage with different infection statuses were selected and placed onto each section of the leaf [31]. The number of eggs laid was counted daily, starting on the first day the female mite began to lay eggs. After laying eggs for five consecutive days, the female mite was removed. The daily egg-laying count and the total number of eggs laid were recorded. Once the eggs hatched into larvae, the hatching rate was recorded, and when they developed into adult mites, the sex ratio (female/male) was noted.

Sexual reproduction: Four different strains of *T*. *turkestani* were selected using different crossbreeding combinations to conduct hybridization experiments. Fresh leaves were divided into four circular sections, each approximately 4 cm^2^. A single female and male mite in the static III stage with different infection statuses were placed together in each section of the leaf, with one pair per section. Two days after the female molted into a mature adult, the male was removed. Starting from the first day of egg-laying, the female mites were removed after laying eggs for five days. The daily egg-laying count and the total number of eggs laid were recorded. After the eggs hatched into larvae, the hatching rate was recorded, and the sex ratio (female/male) was noted once the mites reached adulthood. If the parental male adult mite died before the female mite started laying eggs, it was promptly replaced with another male adult mite. If the parental female adult mite died before completing five days of egg laying, the data for that pair were discarded. The cytoplasmic incompatibility (CI) level (CI%) was calculated using the following formula: CI% = (1 − F/FC) × 100, where F represents the number of female offspring from incompatible crosses (♀Iu × ♂I_W_, ♀Iu × ♂I_WR_), and FC is the average number of female offspring from the control cross (♀Iu × ♂I_U_) [34]. The embryonic mortality (EMs) of different *T*. *turkestani* strains (parthenogenetic individuals or sexually reproduced individuals) was calculated using the following: formula EM = TE − HE, where TE is the total number of eggs in a single cross, and HE is the number of hatched eggs. The post-embryonic mortality (PEM) of each crossbreeding combination (♀I_U_ × ♂I_WR_, ♀I_U_ × ♂I_W_) was calculated using the following formula: PEM% = (1–AO/HE) × EM, where AO is the number of adult offspring in a single cross [35].

The above experiments were repeated 30 times. Under a microscope, the number of eggs laid by a single female mite or each pair of parents was counted, and the number of embryonic deaths and nymph deaths was recorded. Adult mites were collected for gender identification.

### 2.8. Data Processing

One-way analysis of variance (SPSS 26.0) was used to compare the outputs of arrhenotokous parthenogenesis and bisexual reproduction in the I_WR_, I_W_, I_R_, and I_U_ strains, and to analyze the CI function of *Wolbachia* in the I_WR_ and I_W_ strains. Pairwise comparisons of all variables were performed using Duncan’s multiple range test. Independent sample t-tests (SPSS 26.0, *p* < 0.05) were employed to analyze the infection titers of *Wolbachia* and *Rickettsia* in *T. turkestani* of different genders in the I_WR_, I_W_, and I_R_ strains, to compare the outputs of arrhenotokous parthenogenesis and bisexual reproduction in the I_WR_, I_R_, and I_W_ strains, and to analyze the impact of male killing induced by *Rickettsia* on the CI function induced by *Wolbachia*. Graphpad Pism9.5 was used for graphing.

## 3. Results

### 3.1. Diagram of the Life Cycle of T. turkestani and Phylogenetic Analysis of Wolbachia and Rickettsia

A phylogenetic tree was constructed based on the *Wolbachia wsp* sequence and 25 *Wolbachia* strains from different species in the database, and the *Wolbachia*-infected *T. turkestani* were classified into group B. The *Wolbachia* infecting *T. turkestani* was found to have a relatively close evolutionary relationship with the *Wolbachia* infecting *Diaphorina citri*. A phylogenetic tree was also constructed based on the *Rickettsia gltA* sequence and 19 *Rickettsia* strains from different species in the database. The *Rickettsia* infecting *T. turkestani* was found to have a relatively close evolutionary relationship with the *Rickettsia* infecting *Leptotrombidium* and *Ceutorhynchus*.

### 3.2. Analysis of the Maternal Inheritance Efficiency of Wolbachia and Rickettsia

The maternal inheritance efficiency of the two symbiotic bacteria was determined through the infection rates of *Wolbachia* and *Rickettsia* in the offspring of *T. turkestani*. The results show that in all male and female offspring from the I_WR_, I_W_, and I_R_ lines (Table 1), regardless of whether they originated from parthenogenesis or sexual reproduction, *Rickettsia* and *Wolbachia* were transmitted from the mother with complete infection (100%).

### 3.3. Detection of the Titers of Wolbachia and Rickettsia in T. turkestani

**Figure 2:**
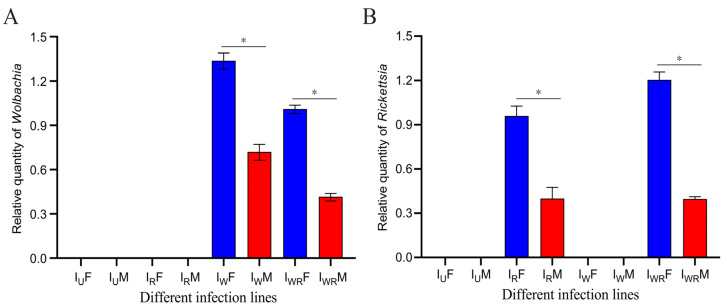
Relative abundance of *Wolbachia* and *Rickettsia* in female and male *Tetranychus turkestani* of different infection strains. (**A**) Relative abundance of *Wolbachia*. (**B**) Relative abundance of *Rickettsia*. I_U_F: female I_U_ population, IuM: male Iu population, I_R_F: female I_R_ population, I_R_M: male I_R_ population, IwF: female Iw population, IwM: male Iw population, I_WR_F: female I_WR_ population, I_WR_M: male I_WR_ population. The symbol “*” indicates a statistically significant difference between the two groups (*p* < 0.05). All error bars represent the standard errors of the means.

The target bands were obtained by PCR amplification using *Wolbachia*-specific primers WSP-236F/44R and *Rickettsia*-specific primers RICTG—F/R (see Supplementary Materials, Figure S1). The I_WR_ strain of *T*. *turkestani* was co-infected with both endosymbiotic bacteria, the Iw strain was infected only with *Wolbachia*, the I_R_ strain was infected only with *Rickettsia*, and the Iu strain was not infected with either of these two symbiotic bacteria. Real-time quantitative PCR was used to measure the titers of *Wolbachia* and *Rickettsia* in male and female adult mites of different strains. The results show that there were significant differences in the contents of *Wolbachia* and *Rickettsia* between the male and female adult mites, with the female adult mites having significantly higher content than the male adult mites. The content of *Wolbachia* in the I_W_ strain was significantly higher than that in the I_WR_ strain (Figure 2A). In contrast, the content of *Rickettsia* was the opposite, with the content in female mites of the I_WR_ strain being higher than that in the I_R_ strain (Figure 2B).

### 3.4. Effects of Different Endosymbiont Infections on the Parthenogenesis of T. turkestani

**Figure 3:**
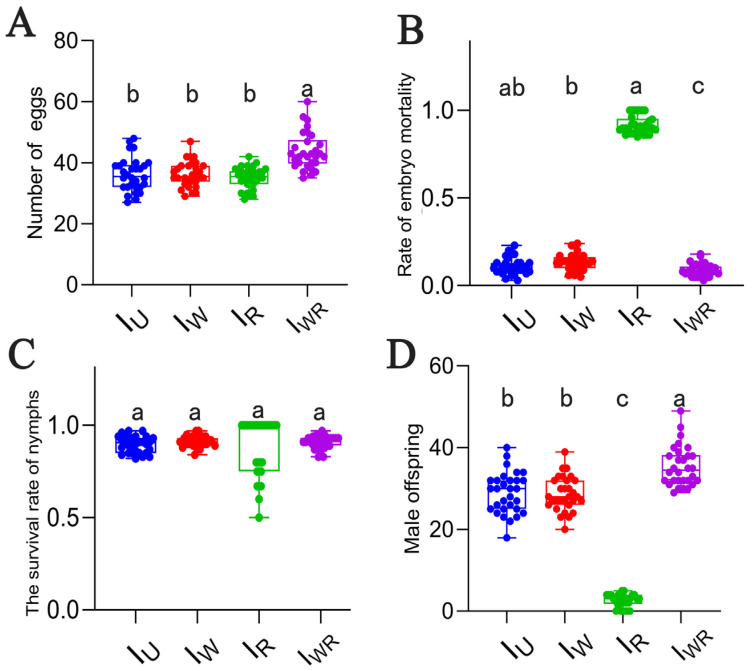
The parthenogenetic parameters of I_U_, I_W_, I_R_, and I_WR_ in *Tetranychus turkestani*. (**A**) Number of eggs. (**B**) Rate of embryo mortality. (**C**) Survival rate of nymphs. (**D**) Male offspring. The data in the figure are the means ± standard errors. The mean values marked by different letter are statistically significant (*p* < 0.05).

Four strains of *T*. *turkestani* reproduced parthenogenetically, with all offspring being male. The average number of eggs laid (per female) of the I_WR_ strain was significantly higher than that of the Iu, I_W_, and I_R_ strains (numbers of eggs laid: 43.80 ± 6.04 vs. 36.20 ± 5.47, 36.20 ± 4.16, and 35.10 ± 3.56, respectively, *p* < 0.001; Figure 3A). The I_WR_ strain had the lowest embryonic mortality rate, while the I_R_ strain had the highest (0.92 ± 0.05, *p* < 0.001, Figure 3B). There were no significant differences in the nymph survival rates among the four strains (Figure 3C). The number of male offspring in the I_WR_ strain was the highest, and that in the I_R_ strain was the lowest. The numbers in the I_U_ and I_W_ strains were at an intermediate level and significantly different from those in the I_WR_ and I_R_ strains (35.47 ± 4.89 vs. 2.50 ± 1.57, 28.97 ± 5.04, 28.67 ± 4.20, *p* < 0.001, Figure 3D). Eggs produced by parthenogenesis from the *T*. *turkestani* strain singly infected with *Rickettsia* failed to hatch normally, and a large number of male embryos died, leading to the lowest number of male offspring in the I_R_ strain.

### 3.5. Effects of Different Endosymbiotic Bacterial Infections on the Sexual Reproduction of T. turkestani

**Figure 4:**
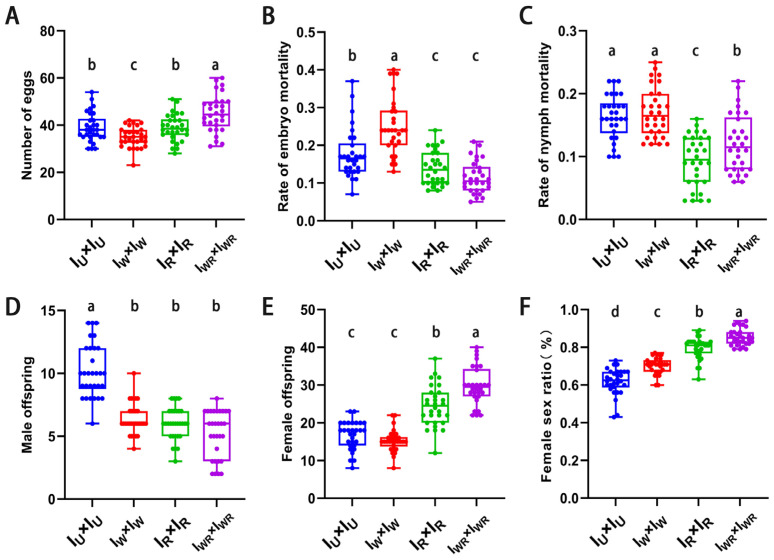
The sexual reproductive parameters of I_W_, I_R_, I_WR_, and I_U_ mating in *Tetranychus turkestani*. (**A**) Number of eggs. (**B**) Rate of embryo mortality. (**C**) Rate of nymph mortality. (**D**) Male offspring. (**E**) Female offspring. (**F**) Female sex ratio. The data in the figure are the means ± standard errors. The mean values marked by different letter markers are statistically significant (*p* < 0.05).

In all four strains, the mated females produced both female and male offspring. The fecundity of the mated female mites of the I_WR_ strain was the highest (45.03 ± 7.78 vs. 38.97 ± 6.13, 34.93 ± 4.32, 38.90 ± 5.63, *p* < 0.013, Figure 4A). Both the embryonic and nymphal mortality rates of the I_WR_ strain were significantly lower than those of the I_W_ and I_U_ strains (embryonic mortality rate: 0.11 ± 0.04 vs. 0.18 ± 0.07, 0.25 ± 0.08, *p* < 0.001, Figure 4B; nymphal mortality rate: 0.12 ± 0.05 vs. 0.16 ± 0.04, 0.17 ± 0.04, *p* < 0.001, Figure 4C). The number of female offspring and the female-to-male sex ratio in the I_WR_ strain were significantly higher than those in the other three strains (number of female offspring: 29.80 ± 5.01 vs. 16.70 ± 3.74, 15.30 ± 2.89, 24.43 ± 5.39, *p* < 0.001, Figure 4E; female-to-male sex ratio: 0.85 ± 0.04 vs. 0.62 ± 0.07, 0.70 ± 0.04, 0.80 ± 0.06, *p* < 0.001, Figure 4F). However, at the same time, the number of male offspring in the I_WR_ strain was significantly lower than that in the I_U_ strain (5.20 ± 1.88 vs. 10.17 ± 2.10, *p* < 0.001, Figure 4D). This indicates that in *T*. *turkestani* co-infection with *Wolbachia* and *Rickettsia*, male offspring die, resulting in an increase in the female-to-male sex ratio. In other words, co-infection with *Wolbachia* and *Rickettsia* can induce male death in *T*. *turkestani*.

### 3.6. Verification of the Male-Killing Effect of Rickettsia in T. turkestani

**Figure 5:**
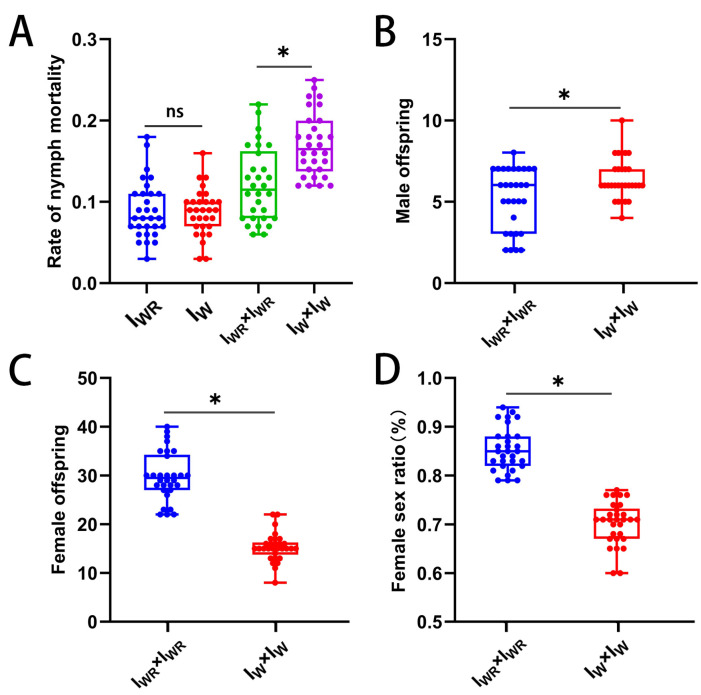
Parthenogenesis and intraspecific sexual reproduction of I_WR_ and I_W_ in *Tetranychus turkestani* within 5 days of oviposition. (**A**) Rate of nymph mortality. (**B**) Male offspring. (**C**) Female offspring. (**D**) Female sex ratio. The data in the figure are the means ± standard errors. The star over the bars “*” represents significant differences between each other at *p* < 0.05, and “ns” represents no significant differences.

The parthenogenesis and sexual reproduction of female mites in the I_WR_ and I_W_ strains were further investigated to verify the male-killing effect of *Rickettsia*. Both the I_WR_ and I_W_ strains produced male offspring parthenogenetically, and there was no significant difference in the nymphal mortality rate of the offspring (Figure 5A). Intraspecific sexual reproduction of female mites in the I_WR_ and I_W_ strains simultaneously produced both female and male offspring. The results show that compared with the I_W_ strain without *Rickettsia*, the I_WR_ strain with *Rickettsia* had a significantly lower nymphal mortality rate (0.12 ± 0.05 vs. 0.17 ± 0.04, *p* < 0.001, Figure 5A). Compared with the I_W_ strain, the number of male offspring in the I_WR_ strain was significantly reduced (5.20 ± 1.88 vs. 6.40 ± 1.25, *p* < 0.01, Figure 5B), while the number of female offspring was significantly increased (29.80 ± 5.01 vs. 15.30 ± 2.89, *p* < 0.001, Figure 5C), and the female-to-male sex ratio in the I_WR_ strain was significantly higher than that in the I_W_ strain (0.85 ± 0.04 vs. 0.70 ± 0.04, *p* < 0.001, Figure 5D). It can be inferred that infection with *Rickettsia* led to the death of more male offspring, resulting in an increase in the female-to-male sex ratio. This verifies that *Rickettsia* induced male killing in *T*. *turkestani.*

### 3.7. Verification of the Cytoplasmic Incompatibility (CI) Induced by Wolbachia in Singly-Infected T. turkestani

**Figure 6:**
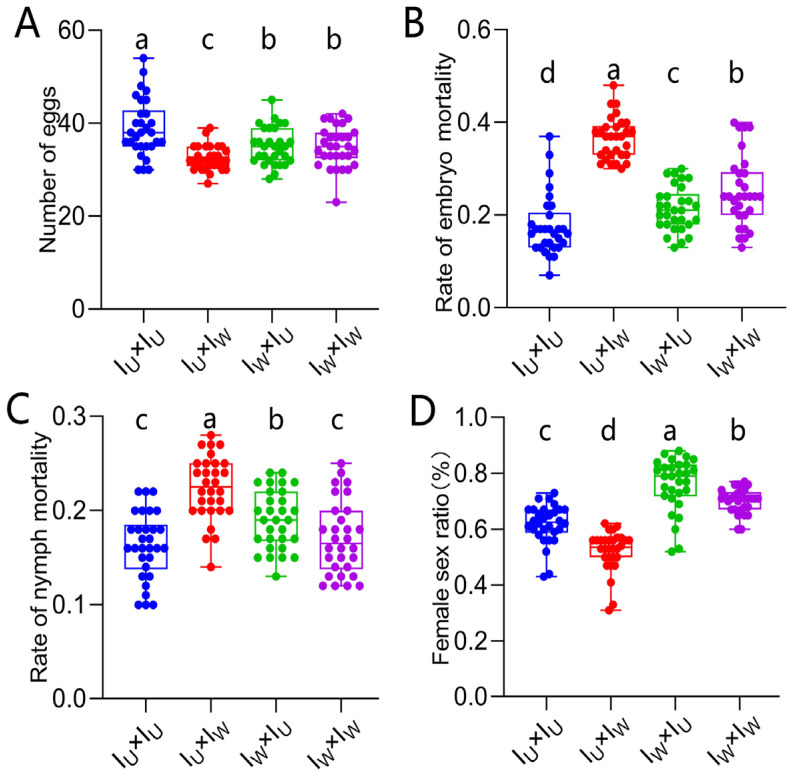
CI identification of Wolbachia in Iw of *Tetranychus turkestani*. (**A**) Number of eggs. (**B**) Rate of embryo mortality. (**C**) Rate of nymph mortality. (**D**) Female sex ratio. The data in the figure are the means ± standard errors. The mean values marked by different letters are statistically significant (*p* < 0.05).

A hybridization experiment using the I_U_ and Iw strains was carried out to verify that *Wolbachia* induced CI in singly-infected *T*. *turkestani*. Among the four different mating combinations, the fecundity of the ♀I_U_ × ♂I_W_ combination was significantly lower than that of the other three combinations (Figure 6A). The embryonic and nymph mortality rates of the ♀I_U_ × ♂I_W_ combination were significantly higher than those of the other three combinations (embryonic mortality rate: 0.37 ± 0.05, *p* < 0.001, Figure 6B; nymph mortality rate: 0.22 ± 0.03, *p* < 0.001, Figure 6C), and the female to male sex ratio was the lowest (0.52 ± 0.07, *p* < 0.002, Figure 6D). These results all indicate that *Wolbachia* induced strong CI in *T*. *turkestani.*

### 3.8. Verification of the Cytoplasmic Incompatibility (CI) Induced by Wolbachia and Rickettsia in Co-Infected T. turkestani

**Figure 7:**
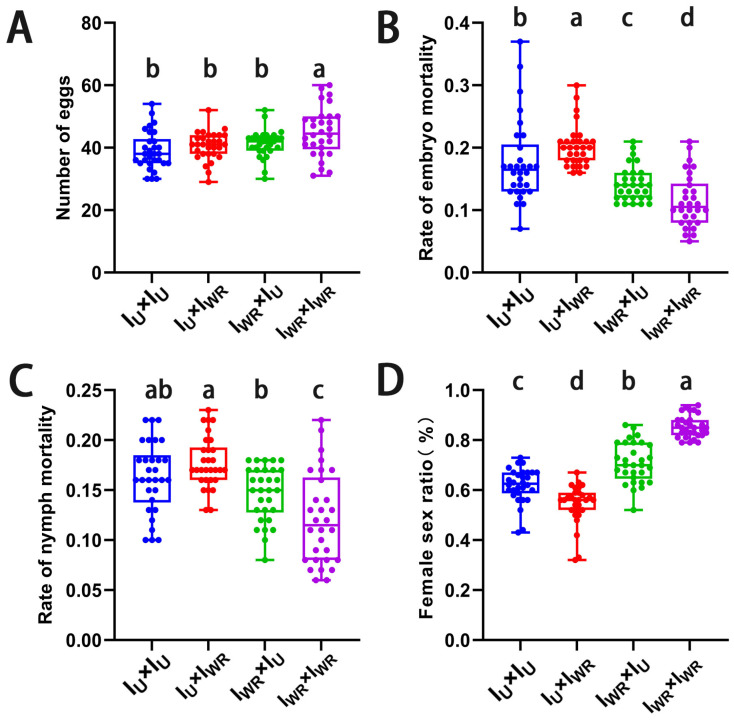
CI identification of Wolbachia and Rickettsia in I_WR_ of *Tetranychus turkestani*. (**A**) Number of eggs. (**B**) Rate of embryo mortality. (**C**) Rate of nymph mortality. (**D**) Female sex ratio. The data in the figure are the means ± standard errors. The mean values marked by different letters are statistically significant (*p* < 0.05).

A hybridization experiment using Iu and I_WR_ strains was conducted to verify that *Wolbachia* and *Rickettsia* induced CI in co-infected *T*. *turkestani*. The fecundity of the ♀Iu × ♂I_WR_ combination was lower than that of the ♀I_WR_ × ♂I_WR_ combination (40.57 ± 4.53 vs. 45.03 ± 7.78, *p* < 0.004, Figure 7A), but there was no significant difference compared with the other two hybridization combinations. The embryonic and nymph mortality rates of the ♀Iu × ♂I_WR_ combination were the highest, significantly higher than those of the ♀I_WR_ × ♂I_WR_ combination (embryonic mortality rate: 0.20 ± 0.03 vs. 0.11 ± 0.04, *p* < 0.001, Figure 7B; nymph mortality rate: 0.18 ± 0.03 vs. 0.12 ± 0.05, *p* < 0.001, Figure 7C). Meanwhile, the female-to-male sex ratio of the ♀I_U_ × ♂I_WR_ combination was significantly lower than that of the other three groups (0.54 ± 0.08 vs. 0.62 ± 0.07, 0.71 ± 0.08, 0.85 ± 0.04, *p* < 0.001, Figure 7D). These results indicate that *Wolbachia* and *Rickettsia* also induced CI in co-infected *T*. *turkestani*.

### 3.9. Antagonistic Effect of Rickettsia-Induced Male Killing on the Strength of Wolbachia-Induced CI

**Figure 8:**
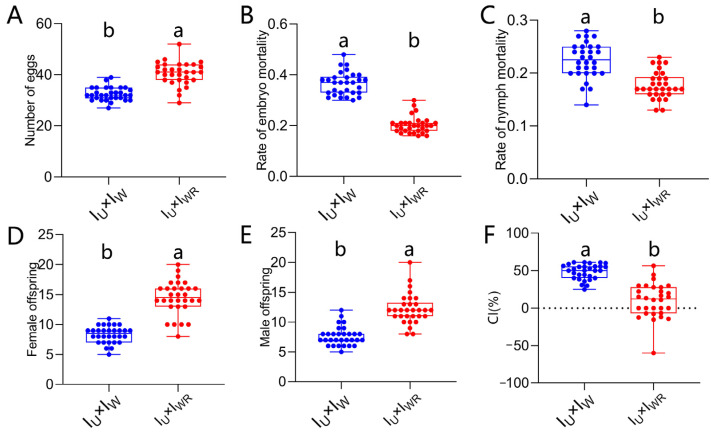
Antagonistic effect of *Rickettsia* co-infection on CI in *Wolbachia*. (**A**) Number of eggs. (**B**) Rate of embryo mortality. (**C**) Rate of nymph mortality. (**D**) Female offspring. (**E**) Male offspring. (**F**) CI%. The data in the figure are the means ± standard errors. The mean values marked by different letter are statistically significant (*p* < 0.05).

After verifying that *Rickettsia* infection can induce male killing in *T. turkestani*, we further investigated the impact of *Rickettsia*-induced male killing on *Wolbachia*-induced CI. Compared with the singly-infected ♀I_U_ × ♂I_W_ combination, the fecundity of the co-infected ♀I_U_ × ♂I_WR_ combination was significantly increased (40.57 ± 4.53 vs. 32.50 ± 2.61, *p* < 0.001, Figure 8A), and the embryonic and nymphal mortality rates were significantly decreased (embryonic mortality rate: 0.37 ± 0.05 vs. 0.20 ± 0.03, *p* < 0.001, Figure 8B; nymph mortality rate: 0.22 ± 0.03 vs. 0.18 ± 0.03, *p* < 0.001, Figure 8C). Compared with the ♀I_U_ × ♂I_W_ combination without *Rickettsia* infection, the co-infected ♀I_U_ × ♂I_WR_ combination had more female and male offspring (female offspring: 14.47 ± 2.80 vs. 8.33 ± 1.40, *p* < 0.001, Figure 8D; male offspring: 12.20 ± 2.54 vs. 7.63 ± 1.59, *p* < 0.001, Figure 8E). Moreover, the CI level of the co-infected ♀I_U_ × ♂I_WR_ combination was significantly lower than that of the singly infected ♀I_U_ × ♂I_W_ combination (9.90 ± 22.77 vs. 48.51 ± 10.13, *p* < 0.001; Figure 8F). This indicates that the I_WR_ strain induced a weaker CI, which might be due to partial antagonism effect of *Rickettsia*-induced male killing on the CI induced by *Wolbachia*.

### 3.10. Wolbachia Does Not Have a Male-Killing Effect on T. turkestani

**Figure 9:**
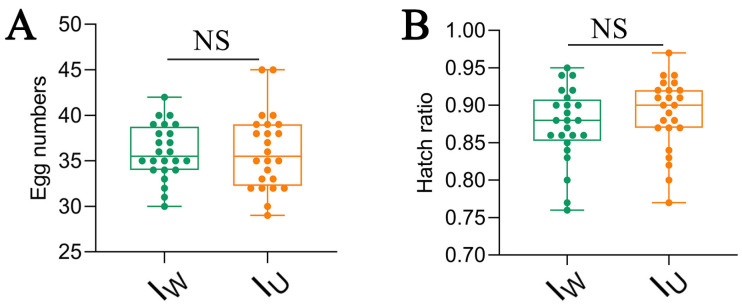
The reproductive parameters of different combinations of *Tetranychus turkestani.* (**A**) Egg production of I_W_ and I_U_ in parthenogenesis. (**B**) Hatching rates of I_W_ and I_U_ in parthenogenesis. I_U_, *Wolbachia*-uninfected; I_W_, *Wolbachia*-infected. Crossing combinations of strains are shown as ‘Female × Male’. The data in the figure are presented as the means ± standard errors. The “NS” represents no significant differences.

Female mites of the I_W_ and I_U_ strains produced male offspring through parthenogenesis, with no significant differences in the fecundity or hatching rate (fecundity: I_W_ vs. I_U_ = 36.00 ± 3.01 vs. 36.08 ± 4.21, *p* > 0.05, Figure 9A; hatching rate: I_W_ vs. I_U_ = 0.87 ± 0.05 vs. 0.89% ± 0.05, *p* > 0.05, Figure 9B). This indicates that *Wolbachia* did not exhibit a male-killing effect on *T. turkestani*.

## 4. Discussion

In this study, different strains of *T. turkestani* infected with different endosymbiotic bacteria (*Wolbachia* and *Rickettsia*) were examined, and phylogenetic analysis was carried out. The results show that the *Wolbachia*-infected *T. turkestani* belonged to supergroup B and could induce cytoplasmic incompatibility (CI) in the host. The transmission efficiency of *Wolbachia* and *Rickettsia* were statistically analyzed. The results reveal that, whether through parthenogenesis or sexual reproduction, both symbiotic bacteria completely followed maternal transmission. Real-time quantitative PCR was used to determine the titers of the endosymbiotic bacteria. The results show significant differences in the abundance of *Wolbachia* and *Rickettsia* between male and female adult mites.

*Rickettsia* is a maternally inherited symbiotic bacterium. In some hosts, it acts as a nutritional symbiont, while in others, it influences the host’s reproduction through reproductive regulations, such as parthenogenesis induction and male killing. It can also enhance the host’s resistance to pesticides and improve the host’s ability to resist predators, high temperatures, or other lethal factors [3,4,17,18,36,37,38,39]. To date, no experimental studies have investigated the reproductive regulation of this bacterium in mites. Our research demonstrates that *Rickettsia*-infected spider mites resulted in parthenogenesis, producing only male offspring, but the hatching rate of male embryos was extremely low. Sexual reproduction in mites singly infected with *Rickettsia* produced both female and male offspring, with a higher number of female offspring than male, resulting in a high female-to-male sex ratio. This indicates that *Rickettsia* infection leads to a male-killing phenotype.

Cytoplasmic incompatibility (CI) is the most common reproductive regulation induced by *Wolbachia* and typically occurs in two forms. The first form is characterized by a high embryonic mortality rate, resulting in a decrease in the number of female offspring, which is called female lethality. The second form does not decrease the total number of offspring but results in an increase in the number of male offspring, known as male development. Both female lethality and male development induced by *Wolbachia* may occur simultaneously in a single insect host, such as the *Wolbachia* wLhetl strain in parasitic wasps [40,41]. In this study, sexual reproduction of *T*. *turkestani* (♀I_U_ × ♂I_W_) significantly increased the mortality rates of embryos and nymphs and significantly decreased the female-to-male sex ratio, indicating the female lethality type. Single infections of *Wolbachia* in *T*. *turkestani* induced strong CI.

Currently, research on the interaction between *Rickettsia* and *Wolbachia* is limited, especially regarding the impact of *Rickettsia* and *Wolbachia* co-infection on the reproductive regulation of host insects, which has not yet been reported. In this study, we found that the *Rickettsia* and *Wolbachia* co-infection induced cytoplasmic incompatibility (CI) in *T*. *turkestani*, but the intensity was much weaker than that induced by single infections of *Wolbachia*. This may be because the male-killing effect of *Rickettsia* in co-infection reduced the CI level induced by *Wolbachia*. Subsequent hybridization experiments clearly demonstrated an antagonistic interaction between the male-killing effect of *Rickettsia* and the CI induced by *Wolbachia*. Previous studies have shown that there are two types of *Wolbachia* in nature: one maintains a high prevalence in insect hosts and weakly induces CI, such as *Wolbachia* in *Drosophila melanogaster* [42]; the other induces strong CI but maintains a low prevalence and titer, like *Wolbachia* in *D*. *melanogaster* [43]. The *Wolbachia* in *T*. *turkestani* studied here was most similar to the former. Furthermore, compared with the control group (I_U_ × I_U_) without a symbiotic bacterial infection, *Rickettsia*–*Wolbachia* co-infection had a higher fecundity, lower mortality, a higher female-to-male ratio, and more offspring. In conclusion, the synergistic effect of the two symbiotic bacteria significantly improved the fitness of *T*. *turkestani*.

**Figure 10:**
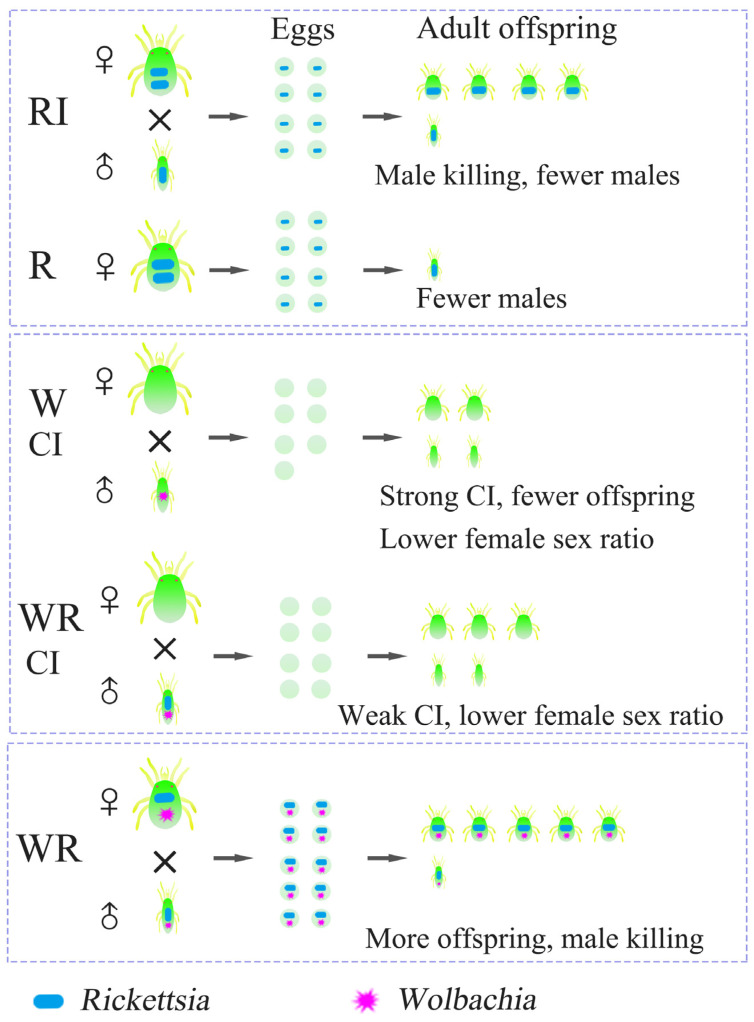
The action model of *Rickettsia* and *Wolbachia* in the reproductive regulation of *Tetranychus turkestani.* RI: *Tetranychus turkestani* with single *Rickettsia* infection; R: parthenogenesis with single *Rickettsia* infection; WCI: cytoplasmic incompatibility (CI) induced by single *Wolbachia* infection; WRCI: cytoplasmic incompatibility (CI) induced by co-infection of *Wolbachia*–*Rickettsia*; WR: *Tetranychus turkestani* with co-infection of *Wolbachia*–*Rickettsia*.

In conclusion, our study reveals five findings (Figure 10): *Rickettsia* infection induced a male-killing effect in the sexual reproduction of *T*. *turkestani*; *Rickettsia* infection caused the death of parthenogenetically produced male embryos in *T*. *turkestani*, leading to a reduction in the number of offspring; *Wolbachia* single infection induced strong cytoplasmic incompatibility (CI) in *T*. *turkestani*, while *Rickettsia–Wolbachia* co-infection induced weaker CI; the male-killing effect induced by *Rickettsia* antagonized the strong CI induced by *Wolbachia*; *Rickettsia–Wolbachia* co-infection promoted the fitness of *T*. *turkestani*, suggesting a synergistic mutualistic relationship between the two symbiotic bacteria within the same host *T*. *turkestani*. We gained a better understanding of the complex interactions between symbiotic bacteria and their hosts, as well as between different symbionts, providing strategies and insights for future applications of symbionts in biological control.

## Figures and Tables

**Table 1:** The maternal inheritance efficiency of *Rickettsia* and *Wolbachia*.

*Tetranychus turkestani* Strains	Number of Adult Female Mites	Number of Offspring	Total Number of Specimens Tested	*Rickettsia*			*Wolbachia*		
				n^+^	n^−^	%	n^+^	n^−^	%
♀I_W_	10	10	100	0	100	0	100	0	100
♀I_R_	50	2	100	100	0	100	0	100	0
♀I_WR_	10	10	100	100	0	100	100	0	100
♀I_W_ × ♂I_U_	10	10	100	0	100	0	100	0	100
♀I_R_ × ♂I_U_	10	10	100	100	0	100	0	100	0
♀I_WR_ × ♂I_U_	10	10	100	100	0	100	100	0	100

Note: n, number; n^+^, number of positive individuals; n^−^, number of negative individuals.

## Data Availability

The original contributions presented in this study are included in the article/Supplementary Material. Further inquiries can be directed to the corresponding author.

## Supplementary Materials

The following supporting information can be downloaded at: https://www.mdpi.com/article/10.3390/microorganisms13030642/s1, Table S1: Symbiotic bacteria sequences in the NCBI database. Table S2: Primer sequences for PCR. Table S3: Gene amplification system. Table S4. PCR amplification procedure. Table S5: Primer sequences for genes. Table S6: Quantitative Real-Time PCR Reaction System. Table S7: PCR amplification procedure. Figure S1: S1 PCR detection of different *Tetranychus turkestani*, *Wolbachia* and *Rickettsia* based on *wsp* and *gltA* genes.
