# Supplementary material for: Interactions Between Endosymbionts Wolbachia and Rickettsia in the Spider Mite Tetranychus turkestani: Cooperation or Antagonism?
Source: Microorganisms. 2025 Mar 12;13(3):642. doi: 10.3390/microorganisms13030642 (PMC11946427; doi:10.3390/microorganisms13030642)
Supplement: Supplementary file 1 [file microorganisms-13-00642-s001.zip › microorganisms-3459692-supplementary.pdf]

Supplementary Materials:

Table S1 Symbiotic bacteria sequences in the NCBI database

Endosymbiont	Host	Accession number	Collection site	Reference
	Tetranychus puerarícola	KU516053	Jiangsu, China	Chen *et al*., 2016
	Tetranychus truncatus	JX094393	Jiangsu, China	Hong *et al*., 2012
	Tetranychus cinnabarinus	DQ016535	Jiangsu, China	Hong *et al*., 2005
	Panonychus mori	AB096234	Ibaraki, Japan	Gotoh *et al*., 2002
	Ctenocephalides felis	KY363325	Xinjiang, China	Zhao *et al*., 2016
	Tribolium confusum	KJ152792	Alberta, Canada	Li *et al*., 2014
	Homalotylus	OL906290	Moscow, Russia	Shaikevich *et al*., 2021
	Bactericera cockerelli	KM267307	Wapato, USA	Cooper *et al*., 2014
	Hyposoter horticola	KF722992	Helsinki, Finland	Duplouy., 2013
	Amphitetranychus viennensis	JX094392	Jiangsu, China	Hong *et al*., 2012
	Drosophila	JX134921	Berkeley, USA	Pantoja and O'Grady,2012
Wolbachia	*Simulium*	MG493290	Glasgow, UK	Baldini *et al*., 2017
	Bemisia tabaci	OL956987	Delhi, India	Ramesh *et al*., 2021
	Chrysotus miripalpus	JQ925541	Zurich, Switzerland	Martin *et al*., 2012
	Podisma sapporensis	JF730716	Novosibirsk, Russia	Ilinsky and Bugrov, 2011
	Macrosteles fascifrons	HQ404761	Jiangsu, China	Hong and Zhang, 2012
	Homalodisca coagulata	DQ450164	Tucson, USA	Takiya *et al*., 2006
	Zygiella x-notata	DQ231507	Norwich, UK	Goodacre *et al*., 2005
	Cubitermes	AY594003	Creteil, France	Harry and Roy, 2004
	Nabis stenoferus	AB109608	Ibaraki, Japan	Kikuchi and Fukatsu, 2003.
	Lasioderma serricorne	KJ152798	Alberta, Canada	Li *et al*., 2014
	Diaphorina citri	OP902291	Guangdong, China	Qiu *et al*., 2014
	Ochlerotatus fluviatilis	KF898395	Belo Horizonte, Brazil	Baton and Moreira, 2013
	Lutzomyia evansi	KM594548	Antioquia, Colombia	Vivero *et al*., 2014
	Tetranychus urticae	AY753175	Gainesville, USA	Hoy and Jeyaprakash, 2005
	Ceutorhynchus	OU906081	Harpenden, UK	King, 2021
Rickettsia	*Adalia bipunctata*	FJ609401	Edinburgh, UK	Weinert *et al*., 2014
	Felis	ON887202	California, USA	Shultz *et al*., 2022

Torix tagoi	AB066351	Ibaraki, Japan	Kikuchi *et al*., 2001
Bulinus truncatus	NR 036948	VIC, Australia	Young, 2022
Adalia decempunctata	FJ609404	Edinburgh, UK	Weinert *et al*., 2009
Acyrthosiphon pisum	U42084	California, USA	Chen, 1995
Leptotrombidium	U17256	Columbus, USA	Fuerst, 1994
Bemisia tabaci	MH908685	Jiangsu, China	Qi *et al*., 2018
Meloidae species	FJ609389	Edinburgh, UK	Weinert *et al*., 2009
Onychiurus sinensis	AY712949	Siena, Italy	Frati *et al*., 2009
Limonia chorea	AF322443	Gent, Belgium	Vandekerckhove, 2000
Neochrysocharis formosa	AB185963	Hiroshima, Japan	Hagimori *et al*., 2004
Dermacentor variabilis	U11014	Columbus, USA	Fuerst, 1994
Adalia bipunctata	U04163	Rochester, USA	Werren, 1993
Kytorhinus sharpianus symbiont	AB021128	Ibaraki, Japan	Fukatsu, 1998
Ixodes scapularis symbiont	D84558	Ibaraki, Japan	Noda, 1996

Table S2 Primer sequences for PCR

Organism	Primer ID	Primers sequences （5-3）	Reference
Rickettsia	RICTG-F	AGGCTAATGGGCTTTGGTCATCGTGTAT	Flore Zélé *et al.*,2018
	RICTG-R	TGTGCCATCCAGCCTACTGTTCTTGC	
Wolbachia	WSP-236F	GACAGTTTAACAGCATTTTCAGGA	赵倩.2019
	WSP-44R	GTTTGATTTCTGGAGTTACATCAT	

Table S3 Gene amplification system

Component	Volume （μL）
2×Taq PCR MasterMIX II	12.5
ddH_2O	9.5
Forward Primer	0.5
Reverse Primer	0.5
DNA	2

Table S4 PCR amplification procedure

Temperature	Time	Number of cycles
95 ℃	2 min	1
95 ℃	30 s	35
55 ℃	30 s	35
72 ℃	45 s	35
72 ℃	5 min	1

PCR amplification was carried out using *Wolbachia*-specific primers WSP-236F/44R and *Rickettsia*-specific primers RICTG-F/R to obtain the target bands. After treatments such as gel recovery, the *Wolbachia wsp* gene fragment was obtained from the target bands. Through NCBI-BLAST alignment, the results showed that the sequence of the symbiotic bacteria *Wolbachia wsp* gene infected by *Tetranychus turkestani* completely matched the sequences with GenBank accession numbers KU516053 and AJ437289. By detecting the infection status of female and male mites of different *Tetranychus* species (including I_{WR}, I_R, I_w, and I_U) during the experimental process, it was found that all species reached complete infection (Figure S1), which can be used for subsequent experiments.

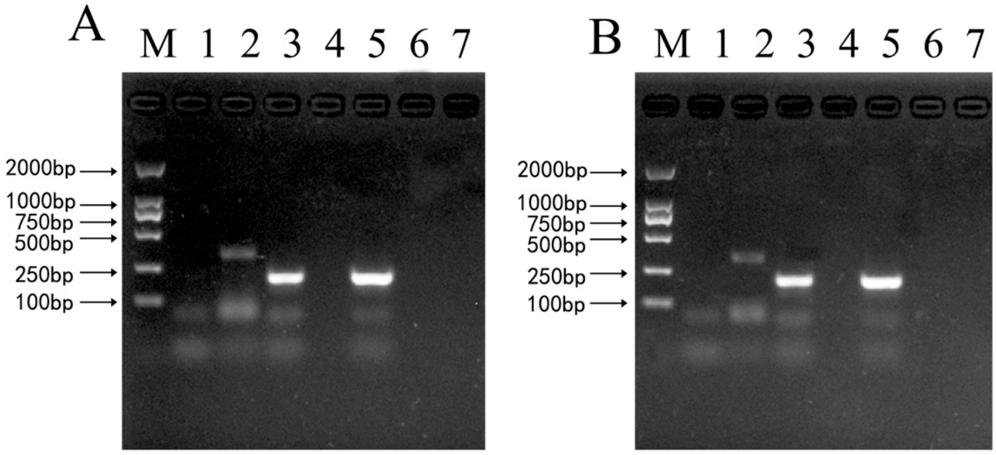

Figure S1 PCR detection of different *Tetranychus turkestani Wolbachia* and *Rickettsia* based on *wsp* and *gltA* genes

Fig A: Female adult of different *Tetranychus turkestani*, Fig B: male adult of different *Tetranychus turkestani*. M: DNA markers, Line 1: ddH_2O, line 2: *Rickettsia* in the I_{WR} population, line 3: *Wolbachia* in the I_{WR} population, line 4: *Rickettsia* in the I_W population, line 5: *Wolbachia* in the I_W population, line 6: *Rickettsia* in the I_U population, line 7: *Wolbachia* in the I_U population.

Based on the *gltA* gene sequence of *Rickettsia* and the *wsp* gene sequence of *Wolbachia*, specific quantitative primers were designed to detect the titers of *Rickettsia* and *Wolbachia* in *Tetranychus turkestani*. The *RPSI8* reference gene was selected as an internal control for data standardization and quantification [25] (Supplementary Tables S5, S6, S7). Adult male and female mites from different infected strains were quantified, with 200-300 individuals per group constituting one replicate, and the experiment was repeated three times. The quantitative PCR (qPCR) reactions were performed on ABI Prism 7500 qPCR instrument. The PCR cycling conditions were as follows: 95°C for 30 seconds; 95°C for 5 seconds, 60°C for 30 seconds, 40 cycles. To verify the specificity of the qPCR products, a melting curve (95°C for 15 seconds, 60°C for 1 minute, 95°C for 15 seconds) was conducted at the end of the reaction. Three technical replicates were performed for each sample. A negative control was set for each reaction. The titer data of Wolbachia and Rickettsia in Tetranychus turkestani were analyzed using SPSS software, and the expression levels were calculated by the $2^{-\Delta\Delta Ct}$ method. The statistical significance analysis was performed using Student's t test.

Table S5 Primer sequences for genes

Organism	Primer ID	Purpose	Primers sequences （5-3）	Reference
Rickettsia	RQ-Fl	qPCR	GCGTTATATTAACGGATGCTTG	⁎
	RQ-Rl	qPCR	TACTTGAAGGGATACCGATAG	
Wolbachia	WQ-F1	qPCR	GATTTCTGGAGTTACATCATAGC	
	WQ-R1	qPCR	GTTGGTGTTGGTGTTGGT	
T. truncatus	RPS18-F	qPCR	ACGTGCTGGTGAACTTACCGAAGA	Sun et al.,2010
	RPS18-R	qPCR	TGCCTATTCAAGAACCAAAGTGGG	

Table S6. Quantitative Real-Time PCR Reaction System

Reagents	Volume
2×PerfectStart Green qPCR SuperMix	10μl
Universal Passive Reference Dye（50×）	0.4μl
Forward Primer（10μM））	0.4μl
Reverse Primer（10μM）	0.4μl
ddH_2O	7.8μl
Template	1μl

Table S7 PCR amplification procedure

Temperature	Time	Number of cycles
95 ℃	30 s	40
95 ℃	5 s	40
60 ℃	30 s	40
95 ℃	15 s	1
65 ℃	1 min	1
95 ℃	15 s	1
